# A novel semiconductor-based, fully incoherent amplified spontaneous emission light source for ghost imaging

**DOI:** 10.1038/srep41866

**Published:** 2017-02-02

**Authors:** Sébastien Hartmann, Wolfgang Elsäßer

**Affiliations:** 1 Institute of Applied Physics, Technische Universität Darmstadt, 64289 Darmstadt, Germany; 2 Center of Smart Interfaces, Technische Universität Darmstadt, 64287 Darmstadt, Germany

## Abstract

Initially, ghost imaging (GI) was demonstrated with entangled light from parametric down conversion. Later, classical light sources were introduced with the development of thermal light GI concepts. State-of-the-art classical GI light sources rely either on complex combinations of coherent light with spatially randomizing optical elements or on incoherent lamps with monochromating optics, however suffering strong losses of efficiency and directionality. Here, a broad-area superluminescent diode is proposed as a new light source for classical ghost imaging. The coherence behavior of this spectrally broadband emitting opto-electronic light source is investigated in detail. An interferometric two-photon detection technique is exploited in order to resolve the ultra-short correlation timescales. We thereby quantify the coherence time, the photon statistics as well as the number of spatial modes unveiling a complete incoherent light behavior. With a one-dimensional proof-of-principle GI experiment, we introduce these compact emitters to the field which could be beneficial for high-speed GI systems as well as for long range GI sensing in future applications.

A “ghost image” is obtained by measuring the total intensity of the transmitted or reflected light of an illuminated object and the spatially resolved intensity of a highly correlated reference beam which itself has never interacted with the object. The information of both intensities alone is not enough to form an image of the object. However, image reconstruction can be achieved by correlating the two intensities. Originally demonstrated with entangled photon pairs from a parametric down-conversion source^1^, the ghost imaging (GI) phenomenon was later shown with classical light^2^ followed by numerous experiments exploiting classical thermal correlations. There, mostly pseudo-thermal light sources based on coherent laser light in combination with a speckle generating diffusive element^3^,^4^ and less often spectrally filtered incandescent lamps were used^5^. Over the past years, a lot of effort has been put into finding GI applications with advantageous imaging properties. GI is promising for imaging through turbulent media^6^,^7^,^8^ and imaging in a harsh environment^9^,^10^. Alongside, computational GI was developed which relies on artificially generated spatially random beam patterns, e.g. by illuminating a spatial light modulator^11^,^12^. Closely connected to computational and compressed sensing methods^13^,^14^, this concept enables single pixel camera systems which considerably reduces the complexity of the detection setup. Moreover, the strive towards a real-world application has led to several proof-of-principle demonstrations such as GI LIDAR^15^, ghost holography^16^, GI microscopy^17^, GI using sunlight^18^, GI with adaptive imaging techniques^19^,^20^, temporal GI^21^,^22^, to name a few.

We choose a different approach to contribute to the applicability of GI, namely by simplifying the light source concept. The requirements for a suitable light source in a classical GI experiment can be summarized to (i) non-constant intensity auto-correlations, i.e. a super-Poissonian photon distribution^23^ and (ii) spatial correlations which are able to resolve a targeted object^24^.

In this article, we show that broad-area superluminescent diodes (BA-SLDs) provide intrinsically all requirements as a GI light source. Owing to the spectrally broadband amplified spontaneous emission, interferometric two-photon-absorption detection is applied in order to work at ultrashort correlation timescales^25^,^26^. A comprehensive coherence analysis of the BA-SLD in operation is given describing quantitatively all properties relevant to GI. A proof-of-principle GI experiment reconstructing the cross-section of a double-slit object is presented, demonstrating its GI capability. The improvement of resolution, the access to two-dimensional GI as well as the potential for future GI sensing applications using BA-SLDs are finally discussed. This work demonstrates the GI phenomenon, for the first time, with a completely incoherent light source.

## The broad-area superluminescent diode

Superluminescent diodes are semiconductor-based opto-electronic emitters capable of emitting spectrally broadband light with several tens to hundreds of nanometer spectral width in terms of wavelength together with high output powers. Therefore, a broadband optical gain material is embedded inside a waveguide structure. To prevent longitudinal mode formation as in laser resonators, the facets are anti-reflection (AR) coated and slightly tilted with respect to the waveguide optical axis. The length of the waveguide, typically several millimeters, allows spontaneously emitted photons to be amplified significantly on their one-way travel towards one of the two output facets generating so-called amplified spontaneous emission (ASE). SLD emission is highly directional which supplies efficient broadband light for practical implementation. SLDs are purely injection-current pumped thus constituting easy-to-handle, miniaturized and robust light sources for a vast field of applications such as optical coherence tomography, distance measurements and optical gyroscopes^27^,^28^,^29^,^30^.

**Figure 1:**
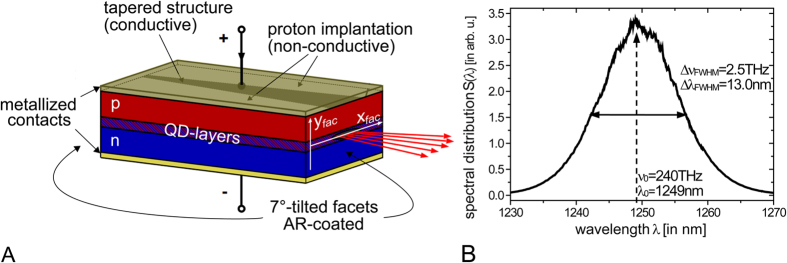
The broad-area superluminescent diode. (**A**) Schematic of the BA-SLD structure. Note that this drawing does not represent the actual proportions. The substrate (n-doped) of the device with about 200 μm thickness is more than 100 times larger than the diode transition. The broad-area facet coordinates are denoted as x_fac_ and y_fac_ perpendicular and along the epitaxial growth direction, respectively. (**B**) Optical spectrum measured with a commercial optical spectrum analyzer with indications of the spectral distribution center and the FWHM both in terms of frequency and wavelength.

The here investigated SLD (see Fig. 1A) is based on a quantum dot (QD) active medium consisting of 15 inhomogeneously broadened InAs/InGaAs QD layers separated by GaAs buffer layers which form a total active layer of 0.620 μm thickness. The 6mm long waveguide is overall tilted by 7° with respect to the facets which are both AR-coated. The tapered waveguide structure consists of a straight section of 500 μm length and 14 μm width followed by the tapered section of 5500 μm length and a resulting facet width of 110 μm. The processing has been made by photolithographic technique and proton implantation inducing a gain-guided waveguide structure in combination with slight index-guiding^31^. Hence, strong amplification with high output powers as well as broad-area (BA) emission at the tapered output facet is implemented. The BA-SLD is operated at room temperature and the pump current is set to approximately 1.3A, well above ASE threshold. An optical spectrum is shown in Fig. 1B revealing near-infrared emission at 1250 nm. The full-width-at-half-maximum (FWHM) amounts to 13 nm which corresponds to 2.5 THz in terms of frequency. The Gaussian-like shape of the spectrum is due to single state (ground state) emission of the optically active QDs^32^.

## Results

### Temporal coherence analysis

This section presents a quantitative temporal coherence analysis of the BA-SLD light. Due to the fact that a THz-large spectral width leads to sub-picosecond correlation timescales, conventional intensity-intensity correlation measurement techniques fail to provide a sufficient time-resolution. Therefore, we rely on an interferometric two-photon-absorption (TPA) detection method^25^. A TPA interferogram I_TPA_(τ) in terms of TPA - photon count rate as a function of auto-correlation time delay τ, is measured within a Michelson configuration. It can be expressed by^33^


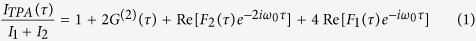


with high-frequency terms F_1_(τ) and F_2_(τ) centered around the mean angular frequency ω_0_ = 2πυ_0_ and 2ω_0_ as well as a low-frequency term G^(2)^(τ) representing the second-order Glauber auto-correlation function^34^. Different temporal correlation orders can thus be analyzed considering the individual terms of Eq. (1) by applying appropriate bandpass filters to the recorded TPA interferogram data (see Methods for more details).

In first place, we consider first-order temporal correlations which are reflected by the F_1_(τ) term. Using the formalism from ref. 33 which assumes a thermal statistics behavior of the light field (see Methods) - we shall see later how well this assumption holds - F_1_(τ) can be directly related to the normalized first-order Glauber auto-correlation function g^(1)^(τ) by


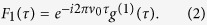


**Figure 2:**
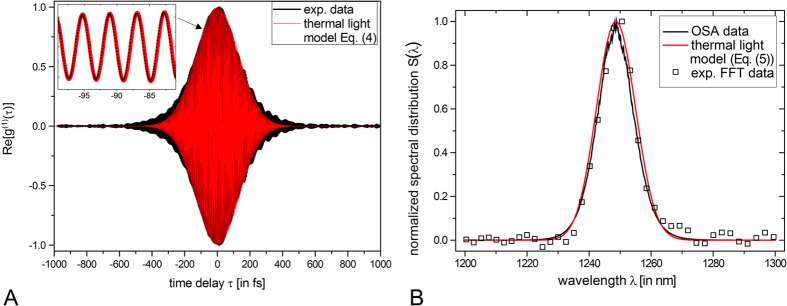
First-order temporal correlations of the BA-SLD light. (**A**) Experimentally determined Re[g(1)(τ)] function (black) and thermal light model with Gaussian distributed optical frequencies calculated with the experimentally determined coherence time τ_c_ = 233 fs (red line); inset: zoom-in showing highly resolved interference fringes; (**B**) optical spectrum obtained by three different methods: an optical spectrum analyzer trace (black line), experimental FFT data (black squares), the Gaussian distribution with τ_c_ = 233 fs (red line).

One can now realize that the fourth term of Eq. (1) is directly proportional to Re[g^(1)^(τ)]. Hence we apply a bandpass filter onto the measured TPA-interferogram using a 40 THz window in order to match the full range of emitted wavelengths (compare with Fig. 1B). The bandpass filtered TPA interferogram is depicted in Fig. 2A (black line). We can exploit the determined Re[g^(1)^(τ)] function to quantify the coherence time τ_c_ defined by^35^


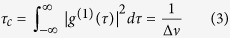


with the spectral width ∆υ in terms of frequency, also called the Suessmann measure^36^,^37^. Given that Re[g^(1)^(τ)] and Im[g^(1)^(τ)] are inherently linked by a Hilbert transform owing to the analytical signal nature of the electric field, we can access |g^(1)^(τ)|^2^ by determining the envelope of (Re[g^(1)^(τ)])^2 ^38. Employing the discrete form of Eq. (3), we obtain a coherence time of τ_c_ = 233 fs which specifies the time window in which correlations of any order are pronounced^39^. Equivalently, we can state that the spectral width, according to the Suessmann definition, amounts to ∆υ = 4.29 THz or ∆λ = c_0_∆υ/υ_0_ = 22.3 nm in terms of frequency and wavelength, respectively. In order to verify this result, we make a comparison with a g^(1)^ function exhibiting Gaussian distributed optical frequencies expressed by^40^


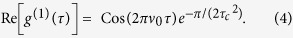


Figure 2A illustrates the excellent coincidence of experimentally determined and thermal light expected Re[g^(1)^(τ)]-functions. Slight deviations in the periphery of the interference fringes are attributed to the limited experimental frequency resolution of 0.5 THz and the non-ideal Gaussian distributed optical spectrum. The latter is explicitly examined in Fig. 2B showing the Fourier transform of the experimental Re[g^(1)^(τ)] (black squares), the measured optical spectrum (black line) as well as the Gaussian distribution S(λ) (red line) corresponding to Eq. (4) which is calculated by the well-known Wiener-Khinchin theorem:


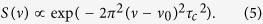


We emphasize the excellent correspondence of all three distributions supporting strongly (i) the accuracy of the implemented measurement system as well as (ii) the correctness of a Gaussian-like spectral distribution of the BA-SLD light.

In order to analyze temporal second-order correlations, we exploit low frequency contributions of the TPA interferogram (Eq. (1) and Methods)^25^:


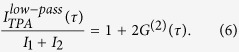


For this purpose, a low-pass filter is applied to the recorded interferogram data, here using a cutoff frequency of 10 THz in the Fourier domain. The normalized second-order auto-correlation function


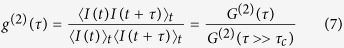


is subsequently calculated with the denominator being derived from the data far off coherence 
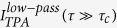
.

**Figure 3:**
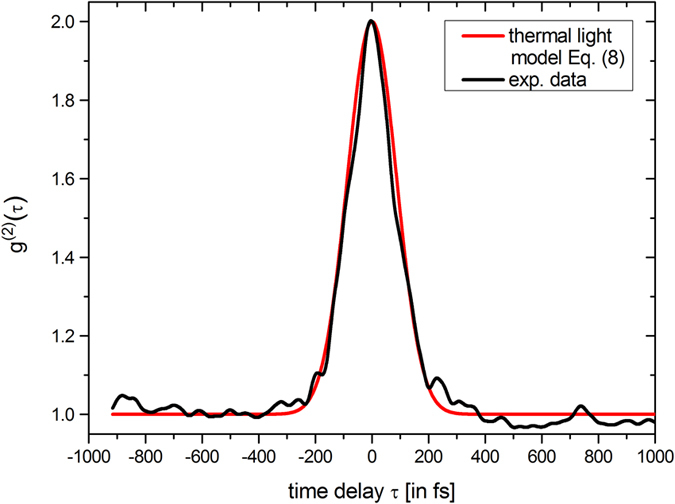
Second-order temporal correlations of the BA-SLD light. Extracted correlation function (black) from a measured TPA interferogram and theoretically expected second-order correlation function for thermal light with Gaussian distributed frequencies calculated with the experimentally determined coherence time τ_c_ = 233 fs (red line).

Figure 3 shows the extracted g^(2)^ function (black line) which exhibits ultrafast decaying correlations dictated by the coherence time of τ_c_ = 233 fs. Most importantly, the central second-order coherence degree g^(2)^(τ = 0) reveals an ideal thermal value of 2.01 ± 0.04. Analogous to the first-order investigation, we compare this experimental result with theoretical expectations assuming an ideal thermal light g^(2)^ behavior based on a Gaussian spectral distribution^40^:


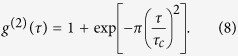


Figure 3 (red line) depicts this function with the experimentally determined value of τ_c_ coinciding excellently with the experimental g^(2)^(τ) data.

Summarizing this section, the temporal coherence properties of the BA-SLD equal literally ideal thermal light behavior for both, first-order correlations revealing femtosecond coherence timescales as well as second-order correlations obeying photon bunching governed photon statistics (see refs 37, 41 and 42 for comparison).

### Spatial correlations

In this part, we investigate the coherence of the BA-SLD light in the spatial domain. Exploiting TPA detection within a spatial HBT configuration allows us to measure the transverse coherence length σ_s_ and to deduce the number of emitted spatial modes. Concurrently, the potential spatial resolution for GI is determined. In fact, the transverse coherence area (~σ_s_^2^) determines the scale on which a targeted object can be resolved in GI^23^ without using compressive imaging techniques^43^.

**Figure 4:**
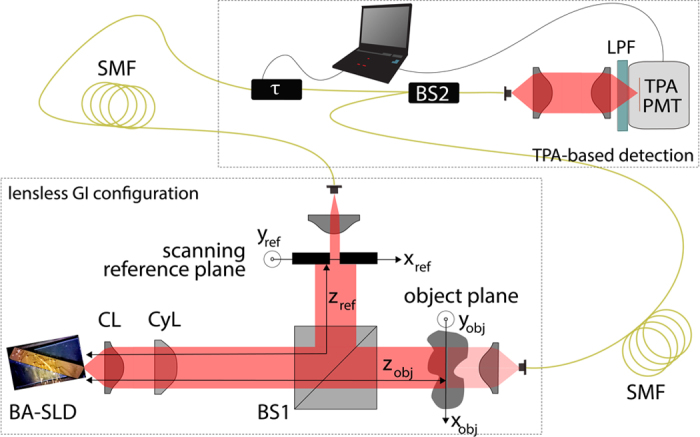
Diagrammatic drawing of the TPA based GI setup. Free-space emitting BA-SLD as the GI light source, collimation lens (CL), cylindrical lens (CyL), two broadband 50:50 beam splitters (BS1, BS2), single-mode fibers (SMF), long pass filter (LPF) blocking fundamental absorptions (λ < 1000 nm, SCHOTT RG1000), and the photomultiplier in TPA mode (TPA-PMT).

Coherent semiconductor laser sources with small area facets typically emit a transverse single-mode, elliptical spatial profile. In contrast, we expect the here employed BA emitter to emit a multitude of spatial modes on the axis perpendicular to the growth direction (x_fac_-axis, Fig. 1A), and a single mode parallel to the growth direction (y_fac_-axis, Fig. 1A). Its dimensions resemble strongly BA laser structures where spatially multi-mode emission and filamentation are well-known phenomena^44^,^45^,^46^. We want to determine σ_s_ at the planes of interest, namely at the imaging planes of a GI experiment. The implemented scheme in Fig. 4 represents such a GI configuration. In a recent publication^26^, the same GI detection scheme was already utilized. However there, the narrow-stripe emitter had to be combined with a rotating ground glass in order to adjust appropriate pseudo-thermal spatial correlations. On the contrary, the here exploited BA-SLD shall serve as a stand-alone GI light source with completely different spatio-temporal mode properties. The emission at the BA facet is collimated by the combination of a short focal length lens (f = 4.5 mm) and a cylindrical lens (f = 100 mm). The first beamsplitter (BS1) creates a statistical copy of the collimated BA-SLD beam and both beams impinge on their corresponding planes, which are both located equidistantly at 600 mm from the source, without magnification. The second-order spatial cross-correlations 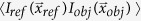 of the light field between the reference plane (x, y)_ref_ and the object plane (x, y)_obj_ are thus equivalent to spatial auto-correlations:


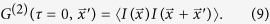


**Figure 5:**
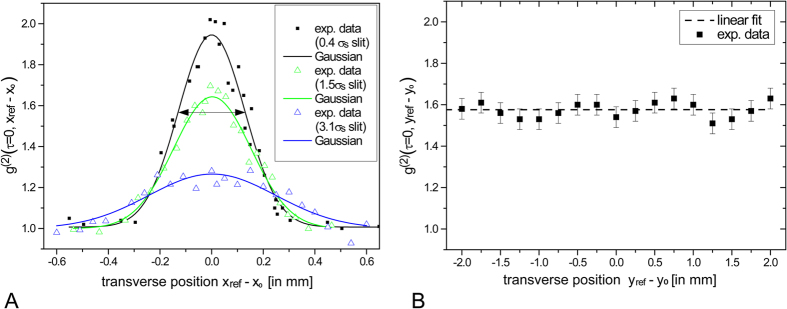
Spatial correlations of the BA-SLD light at the imaging planes. Experimentally determined spatial intensity auto-correlations (**A**) along the horizontal x_ref_-axis with different slit widths (the deduced mode expansion σ_S_ ≈ 2σ_G_ is illustrated by the arrows) and (**B**) along the vertical y_ref_-axis. The latter slope of a linear fit (dashed line) amounts to 0.

〈〉 denotes the average over space 〈〉_x_. At first, we employ slit apertures in each of the two planes with a slit dimensioning of ∆x ∙ ∆y = 100 μm ∙ 4 mm. In order to implement the relative horizontal displacement x + x′, one slit is scanned transversely relatively to the other, here in x_ref_ direction. By recombining the light transmitted through both planes via BS2 and subsequent focusing onto the TPA detector (Fig. 4), G^(2)^(τ = 0) signals are acquired stepwise by measuring a TPA interferogram at each position x_ref_. The result is shown in Fig. 5A (black data) where g^(2)^(τ = 0, x_ref_) is depicted as a function of the transverse position x_ref_. We observe values of g^(2)^ ranging from 1.00 to 2.02. The fact that these values reflect both, no correlation (g^(2)^ = 1) and maximum thermal correlation (g^(2)^ = 2), supports the choice of apertures to describe correct spatial auto-correlations. It also indicates the full linear polarization state of the BA-SLD light. An estimate for the transverse coherence width σ_s_ is given by adapting a Gaussian^23^:


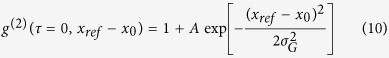


The fit procedure yields an amplitude of 0.94 ± 0.05 and a Gaussian width of σ_G_ = (128 ± 6)μm. The transverse coherence width is approximated by σ_s_ ≈ 2σ_g_^23^ which takes a value of (256 ± 12) μm. In Fig. 5A, the data of two additional measurements is shown where one of the slits is enlarged into ∆x = 375 μm (green data) and ∆x = 800 μm (blue data). One can recognize the typical behavior of contrast reduction and signal broadening when multiple transverse coherence areas contribute to the intensity correlation signal^24^. For ∆x values of 1.5σ_s_ and 3.1σ_s_, the signal amplitudes decrease from 1.94 to 1.64 and to 1.27, respectively. This proves that the implemented GI setup works correctly in the sense that spatial coherence properties are preserved and not affected by e.g. the use of SM fibers. Next, two slit apertures are aligned along the vertical y_ref_-axis with aperture dimensions of (∆x ∙ ∆y) = (1.5σ_s_ ∙ 100) μm^2^. The transverse scan g^(2)^(τ = 0, y_ref_) (Fig. 5B) shows no significant correlation modulation. The overall reduced value from 2 to 1.58 is due to the detection of multiple spatial modes from the horizontal dimension. This measurement thus proves experimentally the transverse single-mode emission in epitaxial growth direction caused by the small dimensioning of 0.62 μm height of the BA-SLD active layer structure. We want to emphasize that the here implemented scheme can be considered as an alternative method for measuring spatial auto-correlations of light sources with ultrafast spatio-temporal mode dynamics, which is specifically challenging when addressing the second-order. Established methods use streak-camera approaches^46^ or first-order interferometry systems^47^.

**Figure 6:**
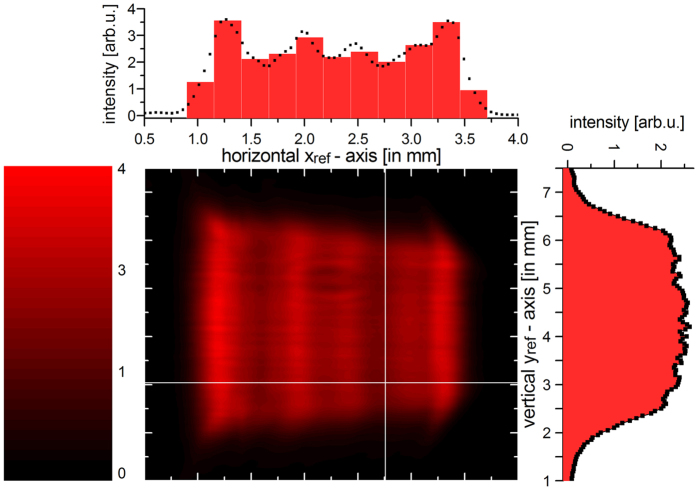
Light spot profile at the imaging planes. The two white lines denote the selected cross-sections depicted as additional graphs at the top and on the right-hand side of the contour plot. In the top graph, the histogram bars illustrate the dominant spatial modes yielding the experimentally determined mode expansion of σ_S_ = 256 μm.

We conclude the spatial coherence analysis by investigating the beam profile in the imaging planes. Figure 6 shows a fiber scanning measurement (62.5 μm core fiber) of the spatial intensity profile of the BA-SLD light in the reference plane. As the GI scheme is lensless, the beam profile in the opposite plane remains identical except of being horizontally inverted. At first, one can see the trapezoid shape of the spot which is characteristic for tapered semiconductor emitters. Secondly, one can recognize the spot dimensions of ~(2.5 × 4.5) mm^2^. Most importantly, the intensity distribution reflects nicely the findings of the spatial coherence properties: On the one hand, a relatively smooth cross-section along the vertical y_ref_-axis is observed reflecting transverse single-mode emission along the growth direction. On the other hand, intensity modulations up to 40% are recorded for the horizontal x_ref_-axis clearly caused by the multiple spatial modes perpendicular to the growth direction of the BA-SLD. From these observations, we can estimate the number of emitted spatial modes exhibiting here a pronounced oblong shape. The number of dominant spatial modes (within a 10 dB range) emitted by the BA-SLD amounts to 11. These modes are indicated in the top cross-section of Fig. 6 as a histogram. As these spatial modes are aligned next to each other along the 110 μm wide BA facet, we can assign an approximate mean near-field mode expansion of 10 μm coinciding well with literature^46^.

### Proof-of-principle ghost imaging experiment

**Figure 7:**
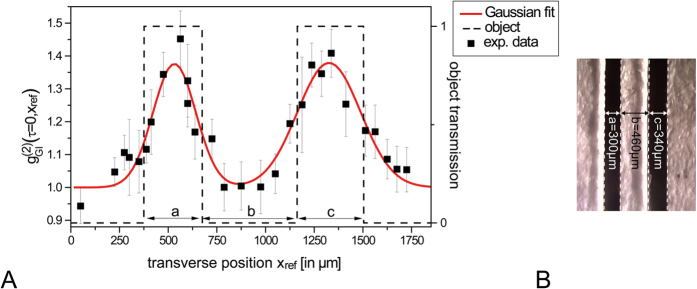
Ghost image cross-section of a double-slit object. (**A**) Experimental data (black squares) with error bars corresponding to the statistical variance of a set of three measurements, fitted double Gaussian function (red line) respecting the error bar weights and indications of the object dimensions (dashed line). (**B**) Digital camera record of the object through a microscope.

The comprehensive coherence analysis showed clear evidence that the BA-SLD yields intrinsically all requirements as a classical GI light source. Even though the here employed device imposes a relatively low image resolution constraint (11 × 1 “pixels”), a proof-of-principle GI experiment using an object of next level complexity can be performed. Therefore, a double-slit object made out of standard reprographic paper and deliberately self-made slits producing an unbalanced object structure (Fig. 7B) is fixed at the object plane within the GI setup. The object and the reference plane are located both at the same distance from the source of z_obj_ = z_ref_ = 600 mm in order to avoid blurring effects. We can moreover specify the detection protocol of Fig. 4 using GI terminology:


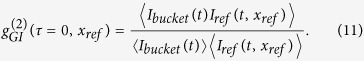


Note that this detection protocol is based on the classical definition of the second-order Glauber correlation function which represents the most basic GI signal detection. The transmitted light through the mask is collected into an optical fiber constituting the bucket intensity I_bucket_(t). The scanning reference arm, comprising a spatially resolving slit of ∆x = 100 μm width and subsequent fiber coupling, acts as the reference intensity I_ref_(t, x_ref_). Here, we restrict ourselves to a one-dimensional space scale as the BA-SLD provides multiple spatial modes solely on the horizontal x-axis.

A cross-section of the double-slit is imaged by the stepwise transverse displacement of the aperture in x_ref_ direction. The obtained values of g_GI_^(2)^ reveal two distinct maxima, g^(2)^(560 μm) = 1.45 and g^(2)^(1340 μm) = 1.41, separated clearly by vanishing correlations of g^(2)^ = 1.0 around x_ref_ = 900 μm. Correlations are completely vanishing at the outer borders of the object at x_ref_ > 1600 μm and x_ref_ < 300 μm. By applying a double Gaussian fit function to the experimental data (Fig. 7A (red line)), we can visualize more image details. Compared to the microscopic picture (Fig. 7B), the ghost image indeed reflects the unbalanced slit widths a = 300 μm and c = 340 μm, the latter featuring a broader FWHM (FWHM_c_ = 380 μm) than for slit a (FWHM_a_ = 260 μm). The slit distance of 780 μm is nicely reproduced reflected by the center position difference of the double Gaussian distribution: s_GI_ = 795 μm. Furthermore, we can derive an overall visibility V = (g_GI_^(2)^_Max_ − g_GI_^(2)^_Min_)/(g_GI_^(2)^_Max_ + g_GI_^(2)^_Min_) of 16%. This good visibility value is achieved at the cost of relatively low image resolution limited by the amount of contributing transverse modes.

## Discussion

We would like to emphasize that this is the first GI experiment with intrinsically fully incoherent light by means of the BA-SLD respecting temporally first-order, temporally second-order as well as spatial incoherence. This semiconductor-based opto-electronic emitter unifies intrinsically all required coherence properties for GI which we analyzed in detail and which are summarized in Table 1. In a recent contribution^26^ we have already exploited the photon bunching properties of ASE emitted by SLDs for GI, however, the spatial coherence had to be adjusted by a rotating diffuser. Here, we exploit the photon bunching and the spatial incoherence of a BA edge-emitter due to the multimode filamentation dynamics along the BA facet coordinate caused by the ultra-fast charge carrier dynamics of semiconductor devices^46^. As there is no need for pre-treatment or post-conditioning of the emitted light, the BA-SLD represents, to the best of our knowledge, the most compact light source in the field. The here presented contribution was enabled by bringing together carefully selected, mature semiconductor emitter technology and recent non-linear intensity correlation detection methods within a GI scheme. Thereby, a completely new type of light source is introduced to the field holding interesting features in view of potential GI applications.

The relatively low image resolution (~256 μm) realized in Fig. 7 (see also Table 1) could be improved in terms of “pixel”-size by either choosing shorter focal-length collimation optics or by refocusing the beam (Fig. 6) onto the object. Here, the near-field expansion of a single transverse mode amounts to ~10 μm. Furthermore, the amount of transverse modes could be increased by designing a BA-SLD with a larger output facet. Also, a straight waveguide instead of the here utilized tapered waveguide would be preferable in order not to impose restrictions on transverse mode generation caused by the small area back facet. GI application specific BA-SLDs could be designed on demand regarding optimal image resolution, optical power and directionality (spot size) properties. Despite their BA facet, BA-SLDs maintain a strong directionality suitable for long range imaging^9^,^10^. We would like to remind that for the here presented experiments, the working distance has been chosen rather arbitrarily. On the one hand, the directional emission of the BA-SLD is well-suited for projecting small light spots at long distances from the source. On the other hand, short working distances of a few mm are well accessible by conceiving miniaturized collimation optics. In view of real imaging applications with complex objects, BA-SLD array structures could considerably scale-up the resolution and also overcome the one-dimensional restriction due to edge-emission. We want to mention that potential opto-electronic emitter alternatives exist such as BA lasers and BA VCSELs, which are established devices in remote sensing applications. Both of these emitters provide enhanced optical power density compared to BA-SLDs due to their laser structure, which is favorable to the intensity-dependent TPA detection. Whereas a BA laser can have similar facet dimensions to BA-SLDs, BA VCSELs could enable two-dimensional imaging via BA surface-emission which can provide a high transverse mode number^48^. Also, random lasers^49^ yielding high power per spatial mode properties could be incorporated within the TPA based GI detection scheme. However, careful selection of laser devices must be taken for sufficiently broadband spectral properties for practical TPA interferometry. Let us finally point out that the provided coherence times as well as the spatial correlation timescales^46^ are orders of magnitudes shorter than in state-of-the-art GI light generation. In combination with the TPA method, this novel classical GI concept provides the image signal as a directly measurable detector current, which principally holds no more limitation for high temporal resolution towards high-speed GI in future work.

## Methods

### Interferometric TPA detection

**Figure 8:**
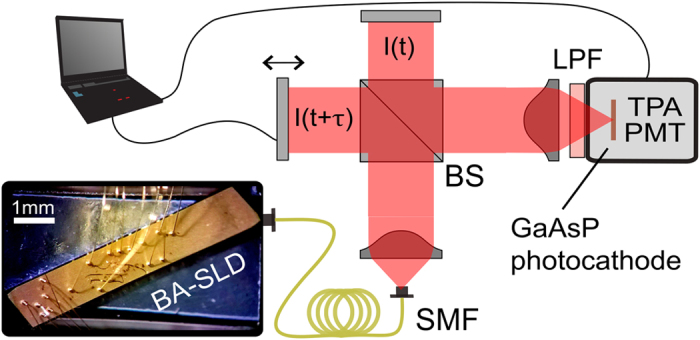
Diagrammatic drawing of the interferometer-based TPA detection for measuring ultra-fast intensity auto-correlations. SMF, single-mode fiber; BS, 50:50 broadband beamsplitter; TPA-PMT, photomultiplier in TPA mode; LPF, longpass filter; Inset: microscopic picture of the BA-SLD device from top.

For the sake of completeness, we want to mention that there exist several techniques to achieve excellent time resolution for g^(2)^(τ)-measurements such as sophisticated time-tagging modules in combination with ultrafast detectors (tens of picoseconds), streak camera approaches (few picoseconds)^51^ and also superconducting detectors (femtosecond regime)^51^. The here employed interferometric two-photon-absorption (TPA) detection technique goes back to the work of Boitier *et al*.^25^ who demonstrated for the first time the photon bunching effect for pure black body sources with multiple THz wide optical spectra. In this context, “pure” means that no spectral filtering but the full range of optical frequencies was measured simultaneously. The nonlinear TPA process requires two photons to be absorbed within a time frame given by the Heisenberg uncertainty Δt ≈ h/4πE_g_^40^,^52^. Hence ultra-fast intensity-intensity correlation detection 〈I(t)I(t)〉 is enabled. By implementing a TPA detector within a Michelson interferometer configuration (Fig. 8), one can introduce a time delay τ to allow intensity auto-correlation measurements 〈I(t)I(t + τ)〉. The photomultiplier in use (Hamamatsu H7421-40) incorporates a GaAsP photocathode (E_g_ ≈ 2.04 eV) which has been selected regarding the source wavelengths in order to guarantee pure TPA photon count detection. By reading out the photon counts from the detector output while varying the optical path of one interferometer arm using a high precision motorized linear translation stage, a TPA interferogram I_TPA_(τ) is recorded. In theory^33^, I_TPA_(τ) comprises four terms (see Eq. (1)): the non-normalized second-order auto-correlation function G^(2)^(τ) = 〈I(t)I(t + τ)〉 and two fast oscillating terms F_1_(τ) and F_2_(τ) following the center angular frequency ω_0_ = 2πυ_0_ and the frequency duplication 2ω_0_ = 4πυ_0_ of the emitted light, respectively. The latter results from the non-linear absorption process.

**Figure 9:**
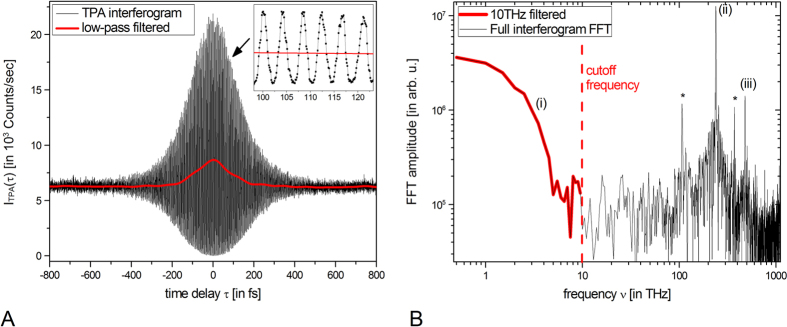
Measurement example. (**A**) TPA interferogram (black) and low-pass filtered data (red) with zoom-in (inset) and (**B**) their corresponding FFTs. Unexpected frequency contributions which cannot be considered as noise but are attributed to interferometric artifacts at the detector are denoted by (*).

In Fig. 9A, the raw data of the recorded TPA interferogram is depicted together with the low-pass filtered signal (Eq. (6)), both as a function of time delay τ = ∆s/c_0_ where c_0_ denotes the speed of light in vacuum and ∆s the optical path delay induced by the displaced mirror. The TPA interferogram reveals on the one hand, enhanced intensity correlations close to τ = 0 reflected by the interferogram asymmetry with respect to the horizontal baseline at 6300 counts per second (C/s) which corresponds to the mean TPA count rate at time delays τ ≫ 0. Figure 9B displays the Fourier transformations (FFTs) of Fig. 9A as a function of frequency υ = ω/2π on a double-logarithmic scale. We can hereby identify the different frequency contributions predicted by Eq. (1): The FFT amplitudes unveil (i) low frequency contribution (υ < 10 THZ) corresponding to the G^(2)^ term, (ii) frequencies attributed to the F_1_(τ) term corresponding to the emitted optical frequencies centered at υ_0_ = 240 THz and (iii) a contribution at 2υ_0_ = 480 THz related to the non-linear F_2_(τ) term. The low-pass signal (Fig. 9A, red line) is extracted by applying an inverse FFT to the low-pass window of Fig. 9B (red data) for calculating G^(2)^(τ) (Eq. (6)) and finally determining g^(2)^(τ = 0), relevant to GI.

### F_1_(τ) extraction

Using the formalism from ref. 33, F_1_ can be expressed generally in a quantum manner by


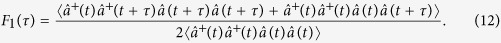


In the case of light with spectral bandwidths ∆υ holding values much smaller than the mean oscillation υ_0_ of the field, one can apply a slowly varying time operator 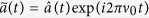 yielding


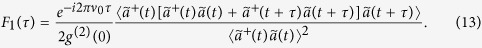


Assuming further a thermal statistics behavior, which is perfectly valid (see Fig. 3), one can apply the well-known Wick theorem^53^ to calculate higher order mean values of combinations of creation and annihilation operators 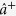 and 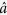, respectively. Appropriate to thermal light, we also insert g^(2)^(0) = 2 resulting with


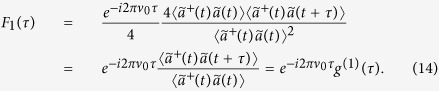


## Additional Information

**How to cite this article:** Hartmann, S. and Elsäßer, W. A novel semiconductor-based, fully incoherent amplified spontaneous emission light source for ghost imaging. *Sci. Rep.* **7**, 41866; doi: 10.1038/srep41866 (2017).

**Publisher's note:** Springer Nature remains neutral with regard to jurisdictional claims in published maps and institutional affiliations.

## Figures and Tables

**Table 1:** Coherence properties of the BA-SLD light.

Coherence	Measure	Impact on optical property
temporal 1^st^ order	τ_c_ = (233 ± 20) fs	(i) ultra-broadband optical spectrum (∆λ = 22.3 nm)
		(ii) fs-timescale correlation decay
temporal 2^nd^ order	g^(2)^(0) = 2.01 ± 0.04	thermal-like photon statistics (i.e. distinct photon bunching)
spatial	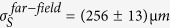 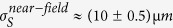	11 × 1 transverse modes
